# Application of High Throughput Technologies in the Development of Acute Myeloid Leukemia Therapy: Challenges and Progress

**DOI:** 10.3390/ijms23052863

**Published:** 2022-03-05

**Authors:** Wei Xiang, Yi Hui Lam, Giridharan Periyasamy, Charles Chuah

**Affiliations:** 1 Department of Haematology, Singapore General Hospital, Singapore 169608, Singapore; xiang.wei@sgh.com.sg (W.X.); lam.yi.hui@sgh.com.sg (Y.H.L.); 2 High Throughput Phenomics Platform, Experimental Drug Development Centre, Agency for Science, Technology and Research (A*STAR), Singapore 139632, Singapore; giri_periyasamy@eddc.a-star.edu.sg; 3 Cancer and Stem Cell Biology Program, Duke-NUS Medical School, Singapore 169857, Singapore

**Keywords:** high throughput, drug screening, AI, leukemia, personalized medicine

## Abstract

Acute myeloid leukemia (AML) is a complex hematological malignancy characterized by extensive heterogeneity in genetics, response to therapy and long-term outcomes, making it a prototype example of development for personalized medicine. Given the accessibility to hematologic malignancy patient samples and recent advances in high-throughput technologies, large amounts of biological data that are clinically relevant for diagnosis, risk stratification and targeted drug development have been generated. Recent studies highlight the potential of implementing genomic-based and phenotypic-based screens in clinics to improve survival in patients with refractory AML. In this review, we will discuss successful applications as well as challenges of most up-to-date high-throughput technologies, including artificial intelligence (AI) approaches, in the development of personalized medicine for AML, and recent clinical studies for evaluating the utility of integrating genomics-guided and drug sensitivity testing-guided treatment approaches for AML patients.

## 1. Introduction

**Figure 1:**
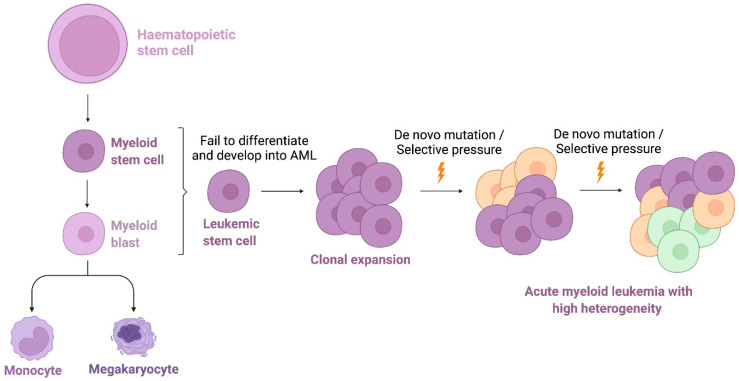
Acute myeloid leukemia (AML). AML is originated from malignant haematopoietic stem cell and is characterized by abnormal clonal expansion and aberrant differentiation of immature clonal myeloid cells. AML progresses with the acquisition of new genetic and/or epigenetic abnormalities in response to chemotherapy and displays substantial heterogeneity. This figure is created in BioRender.com (Available online https://biorender.com/, last accessed on 25 February 2022).

Acute myeloid leukemia (AML) is the most common myeloid neoplasm. The condition is characterized by abnormal clonal expansion and aberrant differentiation of immature clonal myeloid cells (Figure 1). Increasing prevalence, ineffective treatment and poor prognosis of AML are growing concerns, particularly in patients with refractory disease. Clinical management has remained largely unchanged since 1970s. The core therapeutic principles involve the standard regime of multiple courses of induction chemotherapy, allogenic hematopoietic stem cell transplantation or palliative care [1]. However, the treatment outcome varies considerably in the different genetic and biological subtypes of AML. Cytogenetic and sequencing analyses have revealed the complex heterogeneity in de novo and relapsed AML. There are at least 11 genetic and 20 biological subsets, considering differentiation states in leukemic blast cells [2,3]. In addition, deep sequencing has revealed approximately 2000 somatically mutated genes across a 200-patient dataset. In one AML sample, there are more than 400 mutations [4,5]. Several driver mutations have been highlighted. These include NPM1, FLT3, CEBPA, DNMT3A, TET2, RUNX1, ASXL1, IDH1 and IDH2 [6]. Many of the recurrent somatic mutations demonstrate prognostic value and are associated with specific drug targets [2,7,8].

Extensive efforts over the past decades have expanded our knowledge in the functional genomic landscape and molecular events that lead to initiation, progression and resistance of AML. This further adds guidance for treatment decisions to match prognostic factors and treatment responses, that result in better risk stratification and survival outcomes for patients. A small number of targeted therapies aimed at specific mutations and essential molecules involved in leukemogenic pathways has been developed and approved for AML patients with the aim of improving clinical outcomes. These include FLT3 inhibitors (e.g., midostaurin and gilteritinib), CD33 monoclonal antibody (e.g., gemtuzumab ozogamicin), IDH2 inhibitors (e.g., enasidenib), IDH1 inhibitors (e.g., ivosidenib), BCL2 inhibitors (e.g., venetoclax) and hedgehog signalling inhibitors (e.g., glasdegib) [8]. Although these drugs have proven to display initial clinical benefits when combined with conventional therapy, most patients experience disease relapse and ultimately succumb to the disease. This is largely due to conditions where (1) there are more than one driver mutation [9]; (2) disease progresses with the acquisition of new genetic and/or epigenetic abnormalities in response to chemotherapy; (3) substantial heterogeneity exists between AML patients.

There is a shift in therapeutic strategies from conventional combination chemotherapy regimens to a more personalised treatment approach based on individual patient-specific characteristics, i.e., precision medicine. Besides the epidemiological and clinical information, precision medicine largely relies on genetic information provided by high throughput sequencing technologies. A plethora of pre-clinical and clinical studies are currently evaluating the outcomes of genomics-guided, phenomics-guided and genomics-phenomics-guided treatment approaches. This significantly benefits relapsed leukemia patients and those unable to tolerate aggressive chemotherapy [10].

Emerging approaches in precision medicine using high-throughput technologies, such as sequencing analysis and drug screening, play essential roles in generating datasets that are valuable for stratifying patients for treatment and guiding drug discovery. In addition, artificial intelligence (AI) has recently garnered attention in cancer therapy due to its ability in enhancing drug discovery, development and administration [11]. In this review, we will discuss the progress and challenges of using these high-throughput technologies in improving AML patient outcomes, focusing on the development of leukemia therapy.

## 2. High-Throughput Technologies-Based Targeted Therapies

The applications of genomics, epigenomics, transcriptomics, proteomics and metabolomics are attributed to recent advances in next generation genome sequencing, bioinformatics, proteomic analytic tools, along with high-throughput technologies. This is a powerful tool that provides deep and robust data, and has led to a rapid discovery of biomarker candidates in the field of oncology. Furthermore, extended research from these discoveries have provided potential therapeutic targets. This in turn will set a systematic approach to devise strategies to guide therapies, address disease risks, and predict clinical outcomes. The main challenges to bring these assays from bench to bedside are mainly due to high costs, long turnaround times and difficulty in data interpretation.

### 2.1. Integration of Genomice Sequencing

Next generation sequencing (NGS) yielding billions of reads in a single run has allowed unparalleled efficiencies, unlike conventional Sanger sequencing. Using this technology, this has allowed faster interrogation of whole genomes, and extended towards transcriptome sequencing to understand protein profiles and interactions. With the efforts of multiple cancer centres and sequencing laboratories, related patient centric databases have been set up in the public domain. This included the Cancer Genome Atlas Research Network among many others. The database has transformed the understanding of leukemia and uncovered multiple genetic alterations and epigenetic modifications. Deep analysis and prediction models have elucidated information pertaining to the disease molecular subtypes, aid therapy selection, identify targets for improve drug delivery and novel molecular targets in drug discovery.

There is strong evidence that genomic alterations could be identified as molecular targets. One successful example is the identification of all-trans retinoic acid (ATRA) and arsenic trioxide therapy for acute promyelocytic leukemia (APL). The majority (~95%) of APL is characterized by a t(15;17) (q22; q21) translocation resulting in the fusion gene transcript promyelocytic leukemia-retinoic acid receptor alpha (PML-RAR-α). The discovery of this translocation has clinical significance due to its responsiveness to arsenic trioxide and ATRA which promote degradation of PML-RAR-α [12]. ATRA and arsenic trioxide therapy can achieve complete remission in 85% to 90% of patients with not only newly diagnosed but also relapsed APL [13].

Another successful example is the identification of mutations of FMS-like tyrosine kinase 3 (FLT3) receptor in AML [14]. FLT3 mutations occur either as internal tandem duplications (FLT3/ITD, 24% of AML) or as point mutations resulting in single amino acid substitutions within the activation loop of the tyrosine kinase domain (FLT3/TKD, 7% of AML), resulting in a constitutively active kinase [15,16]. Midostaurin is an oral multi-kinase inhibitor with activity against FLT3 kinase. The addition of midostaurin to existing standard-of-care regimes as first line therapy benefits younger AML patients with FLT3 mutations. Midostaurin is approved by USA Food and Drug Administration (FDA) for the treatment of newly diagnosed FLT3-mutated AML [17]. In the management of AML with FLT3 mutations, a number of potential drugs have been investigated including quizartinib (highly selective to FLT3; NCT02039726 and NCT02668653), crenolanib (against both ITD and TKD mutations; NCT02400255) and gilteritinib (potent FLT3/AXL inhibitor; NCT02927262) [18]. Although identification of genetic abnormalities have significantly advanced AML treatment, patients who are not assigned to such specific subtypes do not benefit from genomic sequencing technologies. In addition, most mutations occur infrequently and remain non-targetable.

A significant finding from genomic sequencing in AML is the discovery of mutations frequently localized to genes with epigenetic functions. These include DNMT3A, TET2, IDH1, IDH2, CBP, KAT6A, HDAC2, HDAC3, EZH2, NSD1, ASXL1 and ASXL2 [19]. Epigenetic modifications are similarly important as genetic abnormalities in contributing to AML aggressiveness and more importantly are pharmacologically reversible. Epigenetics regulate gene expression through histone acetylation, and histone and DNA methylation via epigenetic modifiers (e.g., BET, HDAC, DNMT and IDH) [20]. This provides opportunities for targeted treatment using these specific inhibitors. DNMT inhibitors, azacitidine and decitabine, have been approved for AML treatment. These drugs show significant improved response rates in patients when combined with existing chemotherapy [21,22,23]. IDH inhibitors, ivosidenib and enasidenib, also achieved high response rates in refractory AML patients. These inhibitors have recently been approved for IDH-mutated relapsed AML [24,25]. Other inhibitors targeting epigenetic modifiers are either in pre-clinical or clinical stages of development [19].

Although epigenetic drugs display great therapeutic potential by targeting multiple pathways simultaneously, its indication for use is still limited and in its infancy. Larger scale randomized control trials and long-term studies are needed to address the prognostication and predictive capabilities and address any potential adverse events when used in combination with chemotherapy. Various clinical trials are currently ongoing to evaluate genomics-guided treatment approaches in either relapsed or refractory AML. However, the genomic technologies fail to evaluate changes in signalling pathways or proteins that could more directly facilitate risk stratification, predict chemoresistance and aid selection of therapy, independent of mutational status.

### 2.2. Integration of Proteomics

The two most frequently used high-throughput proteomics technologies in leukemia research are mass spectrometry (MS) and antibody-based reverse phase protein array (RPPA), which allow distinguishing protein isoforms, post-translational modifications, and amino acid changes. MS is often used as a de novo discovery platform as it is unbiased towards the protein targets of interest whereas RPPA uses validated monoclonal antibodies to measure protein expression. RPPA is more suitable than MS for direct clinical applications with actual primary samples as it uses less amount of samples. Proteomics technologies used in leukemia studies are helpful to identify protein markers that stratify patients on their prognosis or response to treatment, through comparing protein profiles between leukemia patients and healthy volunteers, between pre and post treatment samples, between drug-resistant and -sensitive cell lines, between leukemia stem/progenitors and bulk cells [26,27,28,29]. For example, BCL2 level is an independent predictor of remission duration and AML patients with high BCL2 expression have shorter overall survival [30]. In addition, this approach could guide therapy selection, via identification of proteins that could facilitate rational drug combination.

Strategies that analyze and integrate net effects of multiple signalling transduction pathways on the entire spectrum of protein effectors and actuators will provide a more complete view of the functions of the leukemic cell population. Large-scale proteomics enable the system-wide characterization of signalling events in the context of functionally related protein groups. This is important as targeting essential signalling pathways has been proven to be effective in improving AML patients’ clinical outcome. BCL-2 is an anti-apoptotic enzyme and prevents leukemia cell death triggered by intrinsic apoptotic mitochondrial pathway [31]. Venetoclax, an inhibitor of BCL-2, has been approved for combination with azacitidine or decitabine or low-dose cytarabine for the treatment of AML patients aged 75 years and above who are not suitable for intensive chemotherapy [32]. Glasdegib, a hedgehog inhibitor is another approved drug used in combination with low-dose cytarabine for treatment of newly-diagnosed AML [33].

The application of proteomics technologies in leukemia has been limited to identification of diagnostic and prognostic biomarkers rather than therapeutic targets in the research laboratories. The critical need is to translate these biomarker discoveries to routine clinical use at an affordable cost. Classification of protein signatures in patients may provide potential drug targets. The challenges to the routine clinical use of proteomics technologies are to develop protein assessment kits that could rapidly enable classification of the protein signature. The ability of utilizing these signatures to evolve as new drugs is also needed [34,35].

### 2.3. Integration of Metabolomics

Similar to genomics and proteomics, metabolic profiling revealed by metabolomics analysis are useful in diagnosis, risk stratification and targeted therapy in AML. The strategy for targeting metabolic activities in AML patients has been employed since cytarabine was used as a standard therapy. Its mechanism of action interferes with nucleotide metabolism [36]. Analytic technologies including proton nuclear magnetic resonance spectroscopy (^1^H NMR) and MS are used as the primary platforms to enable the identification of metabolites and other small molecules in biological samples. Substantial evidence has revealed the unique feature of leukemia cells in the context of cell energy, nucleic acid precursors, amino acids and lipids [37,38,39,40]. The enzymes isocitrate dehydrogenase 1 and 2 (IDH-1 and IDH-2) play critical roles in the citric acid cycle via catalysing the conversion of isocitrate to alpha keto glutarate (αKG). AML patients with IDH1/2 mutations are responsive to ivosidenib (IDH-1 inhibitor) and enasidenib (IDH-2 inhibitor) [24,25]. This illustrates how IDH1/2 are examples of translating metabolomics findings to successful therapeutic targets.

Preclinical and clinical studies highlight the therapeutic potential of several metabolic targets in AML, such as fructose, nucleotide biosynthesis and oxidative phosphorylation [41]. However, it is challenging to achieve a comprehensive characterization of the metabolome. This is largely due to the complexity of metabolite classes and rapid dynamic changes [42]. Standardization of sample preparation and statistical power to account for inter-individual variability are needed for metabolomic studies to maximize sensitivity, specificity and reproducibility. Metabolic interrogation will continue to uncover new insights into AML. However, metabolomics data is complex and metabolites are challenging to annotate. Data reproducibility is another issue due to variability in methods between labs. As a burgeoning field, the gap to be able to adopt metabolomic technology in routine clinical use will need cost reduction of assays and standardization in operating procedures for sample preparation, data processing and analytical platforms.

## 3. High-Throughput Drug Screening-Based Therapies

Although the recurrent genetic/epigenetic/proteomic alterations in acute leukaemia have been linked to actionable targets, identification of these targets in patients using these newer technologies does not always lead to effective therapies. A strategy to circumvent this issue is to use an unbiased drug screening approach, such as high-throughput screening (HTS). HTS plays a pivotal role in the early stages of cancer drug-discovery. The size, quality and content of the compound library are important factors in determining successful HTS screens. Clinically available drug libraries are often used for HTS. Disease models are developed for HTS, including immortalized cell lines, engineered cell systems (eg. reporter cell lines and isogenic cell lines) and co-culture conditions (e.g., cell type 1 vs cell type 2 competition) and patient-derived cells (PDCs).

### 3.1. Drug Sensitivity Testing (DST)

Substantial evidence has shown that DST-guided therapy demonstrates clinical benefits in cancer patients. Chia et al. demonstrated that using monotherapy of gefitinib resulted in significant cancer regression within 6 weeks of treatment in a patient with recurrent oral squamous cell carcinoma [43]. This target was identified as the top candidate through HTS. Swords et al. screened 12 patients with refractory AML using a DST platform with a panel of 215 approved compounds using cell viability assay and evaluated the clinical benefits based on DST results. Among three of four DST-treated patients, treatment responses were achieved. All non-DST-guided patients progressed during treatment [44]. Of note, they demonstrated that DST can be completed in a clinical setting within 10 days which is comparable to standard treatment planning in the non-DST-guided therapy of AML patients [44].

DST on consecutive liquid biopsy samples from the patients allows the physician to rapidly adapt treatment plans in response to drug resistance. Pemovska et al. reported that DST was predictive of clinical response, and therapy based on DST resulted in clinical responses that met the European LeukemiaNet criteria. These included morphological leukemia free state and complete remission [45]. By performing DST on 28 AML and 7 healthy bone marrow samples as normal control using cell viability assay, they assessed leukemia-selective effects of 187 approved drugs by comparing drug effects in AML cells with those of healthy donors. In this study, signal transduction inhibitors such as AML-selective drugs were identified. Although drug sensitivity patterns were heterogeneous, the taxonomy of AML and cancer drugs was established based on the comprehensive drug-response profiles. Monitoring the progression under DST-guided therapies with consecutive DST, and together with mutational and gene expression profiling in patient samples will provide insights into disease evolution, and the relationship between genomic changes and drug response.

Using DST-based HTS platform, a number of recent studies identified combinatory anti-cancer therapies. Drenberg et al. found that gemcitabine and cabazitaxel may be useful for treating pediatric AML [46]. Focusing on a library of natural products, Sweeney et al. identified a synergistic combination of dimethylaminoparthenolide, a sesquiterpene lactone extracted from *Tanacetum parthenium*, and shikonin, a naphthoquinone found in the root of *Lithospermum erythrorhizon*, in pediatric precursor-B cell acute lymphoblastic leukemia [47]. Kurtz et al. performed ex vivo sensitivity profiling of 122 patient samples from myeloid- and lymphoid-derived hematologic malignancies against a panel of 48 drug combinations and identified that the CDK4/6 inhibitor, palbociclib, and the BCL-2 inhibitor, venetoclax displays improved combinatory efficacy [48].

Apart from using microplate readers for readout in DST assay mentioned above, high-throughput flow cytometry has also been used in DST assay. Using 54 patient samples in a large panel of approved and investigational drugs, Spinner et al. demonstrated the utility of a fully automated ex vivo DST platform using high-throughput flow cytometry to identify potential therapeutic drugs for patients with refractory myeloid neoplasms [49]. Drug treated hematopoietic cells were stained with antibodies against CD19, CD3, CD38, CD33, CD34, CD45, CD14, CD66B and HLA-DR prior to high-throughput, multi-parametric flow cytometry to analyse blast viability and differentiation. This platform has been validated to generate results within a clinically actionable time frame and could accurately predict clinical response in vivo [49]. High-throughput flow cytometry has been recently developed in a 96- or 384-well formats to support HTS. Although flow cytometry provides multiparametric analysis and identifies populations of cells in a heterogeneous sample, it has only recently moved into HTS domain. Upgrading existing flow cytometers with HTS functionalities require both hardware and analytical tools [50]. Using a high-throughput flow cytometry-based phenotypic differentiation screen against more than 330,000 small molecules on engineered AML cells, Lewis et al. identified ML390, dihydroorotate dehydrogenase inhibitor, as an inducer of AML differentiation [51].

### 3.2. Data Analysis

To harness the power of high throughput screening, the choice of data analysis methods and subsequent computational approaches are crucial. Initial analysis with the use of readout can range from as simple as relative drug inhibition scores or half maximal effective concentration (EC50) to more complex methods, such as drug sensitivity scoring (DSS). DSS is a recently developed method to quantitatively score differential drug sensitivity for individual anti-cancer therapies. This can be widely applied to experimental settings regardless of technical variability [52]. Compared to IC50 which is a conventional semi-quantitative parameter to indicate drug response, DSS is a more robust scoring approach that integrates multiple dose-response relationships in HTS drug testing studies. Ryall et al. have integrated data from high-throughput drug screening, drug-kinase binding, and transcriptomics using an algorithm named Kinase Addiction Ranker (KAR) [53]. This predicts the dependence of cancer cells to kinases by generating lists of kinases correlated to a specific cellular phenotype i.e., proliferation. KAR has shown that leukemia cells tend to be highly reliant on EPHA5, EPHA3 and BTK kinases, to name a few. This allows potential key cellular targets to be identified and drugged [53].

## 4. Artificial Intelligence (AI) in Cancer Therapy

**Figure 2:**
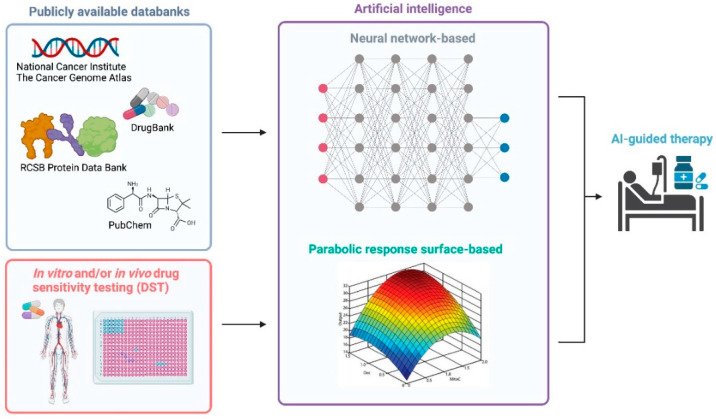
Identification of personalised therapy therapy using artificial intelligence in cancer. Overall framework for the identification of targeted therapy through network-based machine learning. Multidisciplinary databases and drug sensitivity testing (in vitro and in vivo) are used as inputs to train the machine learning model. Parabolic response surface-based map is reproduced with permission from Dr. Edward Chow [56]. This figure is created in BioRender.com (Available online: https://biorender.com/, accessed on 25 February 2022).

AI is poised to disrupt how big data is analysed and will benefit cancer management, including new drug discovery, drug repurposing and optimum dose administration (Figure 2). AI accelerates the process of research, reduces the cost and risk related to clinical trials, harnesses different assays to match patients and aids personalized cancer therapy [11]. Advanced AI-based machine learning plays a pivotal role in targeting novel drugs by analysing and integrating multidisciplinary databases including the Cancer Genome Atlas, DrugBank, PubChem, Protein Data Bank [54]. In addition, AI has successfully predicted drug behaviour, quantified through IC50 values, by using cell genomic features and drug chemical properties [55]. Using an algorithm that tests all possible drug combinations at varying doses, the selection of drug can be made. In each candidate treatment regime, AI aims to identify the best drugs combination, together with the right doses that will result in minimum toxicity or adverse events.

A number of studies have demonstrated that the new AI technology, quadratic phenotypic optimization platform (QPOP), which uses high order polynomial relationships to model complex biological events is very useful in optimizing drug combination therapy design by remarkably reducing the number of experiments needed [56,57]. This platform is able to identify from a pool of possible drug candidates, the drug composition and doses that collectively mediate the best possible treatment outcomes, without reference to molecular mechanisms or predetermined drug synergy data. QPOP successfully predicted an optimal salvage regimen for a patient with refractory lymphoma [57]. Using ex-vivo testing on primary tumor samples followed by QPOP, a highly synergistic interaction between bortezomib and panobinostat was identified. This led to the prediction that the patient would respond better to bortezomib and panobinostat combination than other possible combinations evaluated. After two cycles of bortezomib and panobinostat, the patient achieved complete remission with no evidence of residual disease [57].

CURATE.AI, an AI-derived, indication-agnostic and mechanism-independent technology platform to provide a dynamic dosing optimisation throughout the course of treatment, has been recently developed based on data collected exclusively from the treated individual [58]. Its application for a prospective, single-drug optimization was demonstrated in the management of immunosuppression after liver transplant [59]. Its ability to continually recalibrate personalised profiles was demonstrated to allow for dynamic dose optimisation in a metastatic prostate cancer patient [60].

## 5. A Streamlined Approach of Integrating Genomic Sequencing, HTS-Based DST and AI Technologies for Personalized Treatment in AML

Precision medicine aims to achieve timely tailored medicine to be administered for a particular patient [61]. Treatment options for blood cancer patients are increasingly personalised. In contrast to solid tumors, which requires the expansion of cancer cells from tumor biopsies, blood cancer cells and corresponding normal cells can be easily obtained from peripheral blood or bone marrow for patient-specific drug sensitivity and selectivity screening. PDCs as ex vivo models are advantageous because they can be used to interrogate the efficacy of therapeutics or genetic vulnerabilities in the HTS format. In monitoring disease progression, serial sampling can also be advantageous to address real time changes in disease. HTS can be performed at multiple time points in a relatively short time frame in blood cancer patients to predict the next line of therapy without delaying patient treatment.

HTS-based DST has been recently employed to rapidly identify personalized therapies for blood cancer patients and proved to be clinically implementable [44,49]. Several clinical trials are currently investigating the diagnostic utility and treatment efficacy of DST- and genomic-based precision medicine. In the treatment of 9 relapsed/refractory AML patients based on HTS-based DST, 1 patient achieved complete remission (CR) with minimal residual disease (MRD), 2 showed complete response with incomplete hematologic recovery and 6 had reduced blasts in their bone marrow (NCT01872819). There are currently a number of clinical studies investigating the feasibility of HTS-based DST and genomic data in developing individualized treatment for relapsed/refractory acute leukemia patients, multiple myeloma or plasma cell leukemia (NCT03389347 and NCT02551718).

The Beat AML clinical trial (NCT02927106) demonstrated the integrative use of NGS to assign tailored treatment within 7 days for individual elderly AML patients. This is built on the largest-to-date dataset that integrates sequencing data (2000 most variably expressed genes) from 562 patients and DST data (122 small molecule library) from 363 patients. The dataset encompasses the documentation of clinically relevant gene mutations and allows the identification of potential therapeutics for AML patients [4]. The results show that patients who selected assigned treatment had significant improved overall survival versus those who elected for standard of care treatment. However, the data was not used to stratify patients and the treatment was limited to pre-determined algorithm according to their dataset on cytogenetic, molecular alterations and available targeted therapeutics for specific AML subsets.

**Figure 3:**
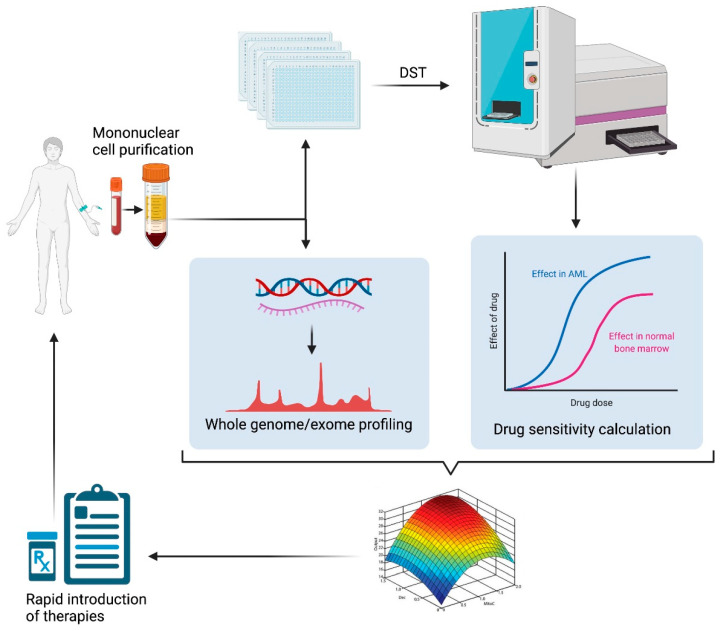
Personalized medicine strategy to tailor treatments for patients with chemotherapy refractory blood cancer. The platform involves (1) in vitro high throughput screening on primary cells from patient samples; (2) deep molecular and genomic profiling of the patients samples; (3) integrating drug sensitivity and sequencing data; (4) optimal drug combination and dosage using experimental-analytic AI platform. Parabolic response surface-based map is reproduced with permission from Dr. Edward Chow [56]. This figure is created in BioRender.com (Available online: https://biorender.com/, accessed on 25 February 2022).

Combination therapies are critical to simultaneously target vulnerabilities in a cancer, and reduce the incidence of therapy resistance. HTS-based DST using PDCs followed by QPOP and CURATE.AI to identify optimal drug combinations and dosing, together with incorporating genomic and transcriptomic profiling to the DST to create a functional taxonomy based on comprehensive drug response and mutation profile will be an important next step toward devising new treatment strategies in blood cancer (Figure 3). This integrative approach will not only advance the understanding of the role of mutational events and specific gene networks in drug response but will also be key in delivering novel therapeutics with optimal combination regimens and dosing to patients.

## 6. Conclusions

Advances in high-throughput technologies have had a positive impact on several facets of personalized medicine in AML and other cancers (Table 1). Several clinical trials have demonstrated the feasibility of implementing genomic and/or drug sensitivity profiling to stratify AML patients for personalized medicine without delaying treatment. However, the utility of these high-throughput technologies for routine clinical use is limited by a number of obstacles. These include the generation of cost-effective high-throughput data, easier and standardized data interpretation, faster turnaround time to make timely clinical decisions, and the need to have a multidisciplinary team. These obstacles can be overcome, in part by using alternative approaches such as QPOP and CURATE.AI. As high-throughput technologies continue to be validated and challenges addressed, its potential to redefine the clinical standards of AML therapy will become evident.

## Figures and Tables

**Table 1:** Pre-clinical and clinical studies integrating high throughput technologies in the development of personalized treatment in AML and other cancers.

Study Name	Approaches	Cancer Type	Outcome	Year	Reference
Ex vivo drug screening defines novel drug sensitivity patterns for informing personalized therapy in myeloid neoplasms	DST-based HTS	MDS	The platform had a positive predictive value of 0.92, negative predictive value of 0.82, and overall accuracy of 0.85.	2020	[49]
Application of an ex-vivo drug sensitivity platform towards achieving complete remission in a refractory T-cell lymphoma	QPOP Co-clinical trial	T-cell lymphoma	Patient achieved CR with an actionable drug combination identified within one week of sample collection	2020	[57]
Ex Vivo Drug Sensitivity Testing and Mutation Profiling	DST-based HTS Genome sequencing	Solid Tumors and Leukemias	Ongoing clinical trial	2019	ClinicalTrials.gov Identifier: NCT03860376
Precision medicine treatment in acute myeloid leukemia using prospective genomic profiling: feasibility and preliminary efficacy of the Beat AML Master Trial	Genome sequencing	AML	Thirty-day mortality was less frequent and overall survival was significantly longer for patients enrolled on the Beat AML sub-studies versus those who elected SOC	2017	[62]
Phenotype-driven precision oncology as a guide for clinical decisions one patient at a time	DST-based HTS Co-clinical trial	head and neck squamous cell carcinomas	Can guide real-time therapeutic decisions	2017	[43]
Beat AML Core Study	genome sequencing	AML	Not available	2016–2020	ClinicalTrials.gov Identifier: NCT02927106
High Throughput Drug Sensitivity Assay and Genomics- Guided Treatment of Patients With Relapsed or Refractory Acute Leukemia	DST-based HTS genome sequencing	AML	Ongoing clinical trial	2015	ClinicalTrials.gov Identifier: NCT02551718
A distinct glucose metabolism signature of acute myeloid leukemia with prognostic value	Metabolomic profiling with GC-TOFMS.	AML	Suggests the use of serum metabolites and metabolic pathways as prognostic markers and potential therapeutic targets for AML	2014	[38]
Global phosphoproteome analysis of human bone marrow reveals predictive phosphorylation markers for the treatment of acute myeloid leukemia with quizartinib.	MS based- phosphoproteome analysis	AML	A signature consisting of five phosphorylation sites predicted the response to quizartinib in AML patients	2014	[26]
Individualized systems medicine strategy to tailor treatments for patients with chemorefractory acute myeloid leukemia	DST-based HTS genome sequencing Co-clinical trial	AML	Can predict clinical responses	2013	[45]
Treatment for Relapsed/Refractory AML Based on a High Throughput Drug Sensitivity Assay	DST-based HTS	AML	Total 9 treated patients 1 CR with MRD 2 CRi	2013	ClinicalTrials.gov Identifier: NCT01872819
Phosphoproteomic analysis of leukemia cells under basal and drug-treated conditions identifies markers of kinase pathway activation and mechanisms of resistance	LC-MS/MS-based phosphoproteomic analysis	AML	Provides valuable information to personalize therapies based on kinase inhibitors	2012	[29]
DIGE-based proteomic analysis identifies nucleophosmin/B23 and nucleolin C23 as over-expressed proteins in relapsed/refractory acute leukemia	DIGE-based proteomic analysis	AML	Upregulation of B23 and C23 could be related to resistance of leukemia	2011	[27]
Identification of prognostic protein biomarkers in childhood acute lymphoblastic leukemia	Proteomic analysis	AML	PCNA as highly predictive of prednisolone response in patients	2011	[28]
